# Extensive sampling and high-throughput sequencing reveal *Posidoniomycesatricolor* gen. et sp. nov. (Aigialaceae, Pleosporales) as the dominant root mycobiont of the dominant Mediterranean seagrass *Posidoniaoceanica*

**DOI:** 10.3897/mycokeys.55.35682

**Published:** 2019-06-26

**Authors:** Martin Vohník, Ondřej Borovec, Zuzana Kolaříková, Radka Sudová, Martina Réblová

**Affiliations:** 1 Department of Mycorrhizal Symbioses, Institute of Botany, Czech Academy of Sciences, Lesní 322, 252 43 Průhonice, Czech Republic Institute of Botany, Czech Academy of Sciences Průhonice Czech Republic; 2 Department of Experimental Plant Biology, Faculty of Science, Charles University, Viničná 5, 128 44 Prague, Czech Republic Charles University Prague Czech Republic; 3 Department of Taxonomy, Institute of Botany, Czech Academy of Sciences, Zámek 1, 252 43 Průhonice, Czech Republic Institute of Botany, Czech Academy Průhonice Czech Republic

**Keywords:** dark septate endophytes, Dothideomycetes, marine fungi, root endophytes, seagrasses

## Abstract

Seagrasses provide invaluable ecosystem services yet very little is known about their root mycobiont diversity and distribution. Here we focused on the dominant Mediterranean seagrass *Posidoniaoceanica* and assessed its root mycobiome at 32 localities covering most of the ecoregions in the NW Mediterranean Sea using light and scanning electron microscopy and tag-encoded 454-pyrosequencing. Microscopy revealed that the recently discovered dark septate endophytic association specific for *P.oceanica* is present at all localities and pyrosequencing confirmed that the *P.oceanica* root mycobiome is dominated by a single undescribed pleosporalean fungus, hitherto unknown from other hosts and ecosystems. Its numerous slow-growing isolates were obtained from surface-sterilised root segments at one locality and after prolonged cultivation, several of them produced viable sterile mycelium. To infer their phylogenetic relationships we sequenced and analysed the large (LSU) and small (SSU) subunit nrDNA, the ITS nrDNA and the DNA-directed RNA polymerase II (*RPB*2). The fungus represents an independent marine biotrophic lineage in the Aigialaceae (Pleosporales) and is introduced here as *Posidoniomycesatricolor***gen. et sp. nov.** Its closest relatives are typically plant-associated saprobes from marine, terrestrial and freshwater habitats in Southeast Asia and Central America. This study expands our knowledge and diversity of the Aigialaceae, adds a new symbiotic lifestyle to this family and provides a formal name for the dominant root mycobiont of the dominant Mediterranean seagrass.

## Introduction

Although the occurrence of marine saprobic and endophytic fungi on mangroves and salt marsh plants is well-documented (e.g. Jones 1963; Jones and Pang 2012; Kohlmeyer and Kohlmeyer 1971; Gessner and Kohlmeyer 1976; Kohlmeyer and Volkmann-Kohlmeyer 1991, 2001, 2002), the mycobiota of seagrasses is generally neglected and relatively little understood (e.g. Kohlmeyer 1963; Kohlmeyer and Kohlmeyer 1979; Cuomo et al. 1985; Alva et al. 2002; Gnavi et al. 2014). Seagrasses are perennial flowering plants represented by several genera inhabiting shore environments practically everywhere outside the Arctic and Antarctic, but mainly in temperate, subtropical and especially tropical littoral zones. All seagrass genera are accommodated in various families of a single order, the Alismatales (Monocotyledons). Unlike most terrestrial and many aquatic plants, seagrasses seem to be devoid of mycorrhizae (Nielsen et al. 1999) and a specific root-fungus association has been so far reported only for a single seagrass species (Vohník et al. 2015).

**Figure 1.**
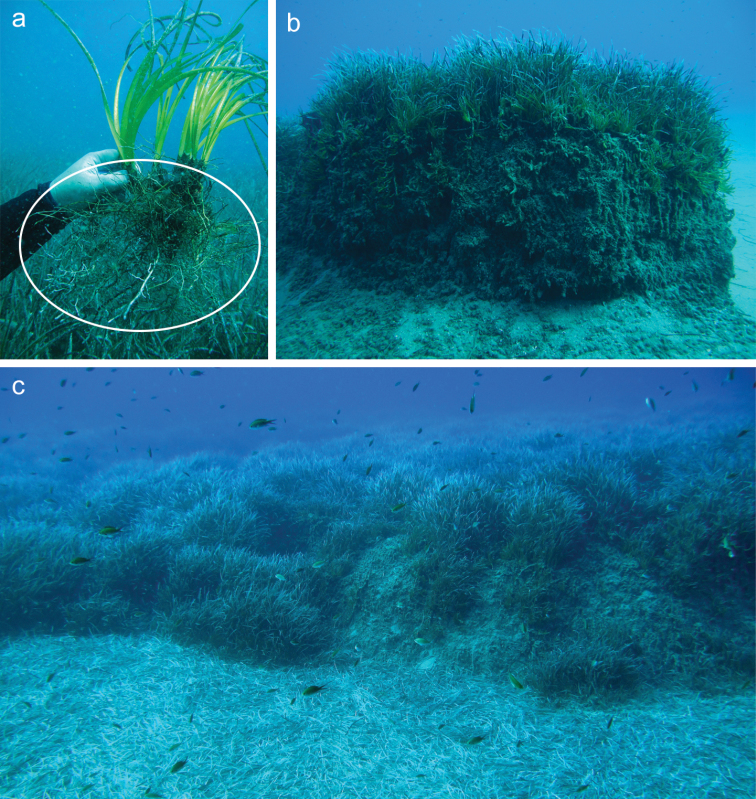
The dominant Mediterranean seagrass *Posidoniaoceanica*. **a** Overall appearance, note dense branched root system of the seagrass (encircled) **b***Posidoniaoceanica* growing on an approx. 1.5 m thick layer of matte **c** typical habitat of the dominant Mediterranean seagrass, note the layer of shed seagrass leaves on the seabed.

*Posidonia* (Posidoniaceae) is the evolutionary oldest seagrass genus with the earliest fossil record from the Cretaceous (den Hartog 1970). It has a uniquely discontinuous distribution with eight of its nine species occurring in the Southern Hemisphere along the coast of Australia (Green and Short 2003). In our study, we focused on root mycobionts of the only non-Australian species, i.e. the dominant and endemic Mediterranean seagrass *Posidoniaoceanica*. In the Mediterranean Sea, *P.oceanica* forms extensive clonal meadows which can be hundreds to thousands of years old and spread over one to several (up to 15) kilometres (Arnaud-Haond et al. 2012). These vast meadows are the primary source of carbon for the coastal ecosystems and, additionally, they play an important role in defining the coastal line and supply biogenic detritus made of seagrass roots, rhizomes and leaf debris entangling other living organisms like molluscs, algae or foraminifera (De Falco et al. 2017). Unlike other seagrasses, *P.oceanica* typically forms extensive branched root systems (Figure 1a) which support formation of “matte” (Figure 1b, c), i.e. a peat-like seabed layer which is exceptionally resistant to microbiological decay and may be up to several metres thick (Hemminga and Duarte 2000; Serrano et al. 2012).

The mycobiota of *P.oceanica* only recently gained appropriate attention; from the few available reports it seems to be predominated by fungi belonging to three classes and five orders of Ascomycota, i.e. Dothideomycetes (Pleosporales, Capnodiales), Leotiomycetes (Helotiales) and Sordariomycetes (Lulworthiales, Microascales and *Papulaspora* incertae sedis). These include obligate marine lignicolous fungi, ubiquitous surface-dwelling saprobes and endophytic fungi colonising roots, rhizomes and leaves, thus forming tighter (symbiotic) relationships with the host plant. Typically, they were growing on living or decaying plant parts (Kohlmeyer 1963; Cuomo et al. 1985) or were isolated as sterile mycelia and identified only by DNA sequence analysis (Panno et al. 2013; Gnavi et al. 2014; Vohník et al. 2016, 2017). They either belong to well-studied genera (e.g. *Corollospora*, *Halotthia*, *Lulworthia* and *Papulaspora*) or represent new marine lineages.

Our previous microscopic observations revealed that living terminal roots of *P.oceanica*, particularly their surface and the thick-walled hypodermis, are regularly colonised by an unknown fungus with dark septate hyphae (Vohník et al. 2015). The resulting association resembles colonisation by the so-called dark septate endophytes (DSE) which regularly occur in the roots of most terrestrial plants (e.g. Jumpponen and Trappe 1998; Vohník and Albrechtová 2011; Lukešová et al. 2015) but seemed to be absent in the marine environment. The association is characterised by the formation of sparse, dark pigmented hyphae, dense finger-like pseudoparenchymatous nets or loose hyphal sheaths on the root surface and melanised intracellular microsclerotia in the hypodermis. However, in contrast to typical terrestrial DSE, although the dark septate hyphae were also infrequently observed inside rhizodermal cells, they never colonised vascular tissues of the host roots. Interestingly, this association was absent in the roots of *Cymodoceanodosa*, a widely distributed seagrass in the Mediterranean Sea which sometimes accompanies *P.oceanica* (Vohník et al. 2015).

In our previous work focused on the diversity and distribution of *P.oceanica* root mycobionts, cultivations and 454-pyrosequencing of fungal DNA from surface-sterilised root segments from a few localities in the NW Mediterranean Sea revealed a relatively narrow fungal community lacking typical terrestrial and freshwater endophytes and mycorrhizal fungi (Vohník et al. 2016, 2017). This unusually limited fungal spectrum (cf. Kohout et al. 2012, 2013; Bruzone et al. 2017) was dominated by a single dark-pigmented mycobiont tentatively named “Pleosporales sp. MV-2012” (Vohník et al. 2016). Interestingly, this symbiotic fungus has not been documented in any of the other studies on *P.oceanica* mycobiota (see above) and to our knowledge it is not known from any other hosts and environments. Its extremely slow growth and characteristic colony morphology enable unequivocal identification already during the isolation stage but spore formation has never been observed (Vohník et al. 2016). Consequently, despite the striking DSE root colonisation pattern in vivo (Vohník et al. 2015), the absence of sexual characters and the lack of formation of conidia and conidiophores in axenic culture, either on agar media (standard or containing salt water) or on surface-sterilised root segments placed on nutrient media, pose a difficulty in estimating precise phylogenetic relationships of this dominant *P.oceanica* root mycobiont. Nevertheless, its preliminary position in the Aigialaceae (Pleosporales, Dothideomycetes), based on sequences of the partial nuclear large subunit (nucLSU) 28S rDNA gene, was discussed in Vohník et al. (2016).

The present study was motivated by the need to confirm the presence/dominance of the pleosporalean DSE fungus in the *P.oceanica* root mycobiota at a much larger scale than previously studied as well as the need for circumscription and precise phylogenetic placement of this mycobiont into the fungal system. Thus, we characterised *P.oceanica* root mycobionts using tag-encoded 454-pyrosequencing at 32 localities in the NW Mediterranean Sea (covering the distribution of *P.oceanica* from its westernmost localities to the boundary between the Western and Eastern Mediterranean basins). We also isolated and characterised *P.oceanica* root mycobionts at the locality where the specific DSE association has been observed for the first time (Vohník et al. 2015). Subsequently, characteristic strains of the Pleosporales sp. MV-2012 were selected for its circumscription based on morphological characters and an analysis of a molecular data set consisting of sequences of the following nuclear markers: nucLSU, nuclear small subunit (nucSSU) 18S rDNA gene and the second largest subunit of the RNA polymerase II (*RPB*2) gene. Additionally, an analysis of the unusually divergent ITS region of nuclear rDNA was performed to screen the possible geographical variability of the dominant *P.oceanica* root mycobiont.

## Materials and methods

### Sampling

**Figure 2.**
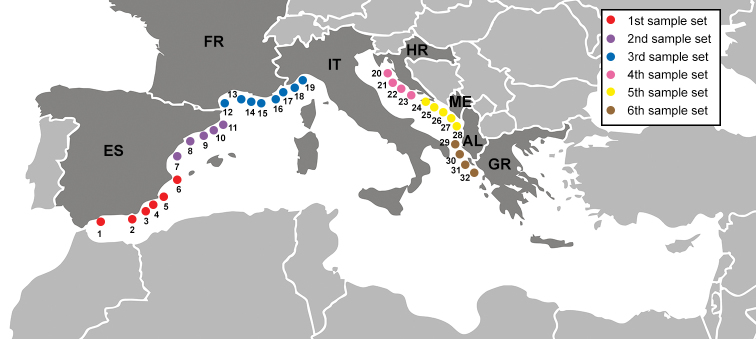
Map of the Mediterranean Sea with location of our 32 sampling sites. For further details see Table 1.

*Posidoniaoceanica* root samples were collected at 32 localities in seven states in the NW Mediterranean (Figure 2) representing four out of the eight Mediterranean Sea ecoregions (Table 1; see Notarbartalo di Sciara and Agardy 2010 in Giacoumi et al. 2013) at various depths using snorkelling and scuba diving. The samples for tag-encoded 454-pyrosequencing were collected in June, July and September 2012 whereas the samples for mycobiont isolation were collected in September 2016 (Table 1). Each locality was represented by a pooled sample consisting of five subsamples taken at least 3 meters apart (see Vohník et al. 2016).

**Table 1.** List of the *Posidoniaoceanica* localities sampled in this study.

Sample set^1^	Locality #^2^	Locality code^3^	Locality name	Locality ecoregion^4^	GPS coordinates	Sampling time
1^st^	1	ES-21	Bahía de la Plata, Estepona	Alboran Sea	36.42749N, 5.12923W	VII/2012
	2	ES-22	Cabo de Gata	dtto	36.72595N, 2.19537W	VII/2012
	3	ES-23	Villaricos	Algero-Provencal Basin	37.26676N, 1.75151W	VII/2012
	4	ES-27	Cope, Calabardina	dtto	37.43672N, 1.48422W	VII/2012
	5	ES-24	Cabo de Palos	dtto	37.63355N, 0.68996W	VII/2012
	6	ES-25	Calp, Cala el Racó	dtto	38.63556N, 0.07124E	VII/2012
2^nd^	7	ES-28	Platja de Capicorb, Torreblanca	dtto	40.20711N, 0.25956E	VII/2012
	8	ES-26	Platja dels Muntanyans, Torredembarra	dtto	41.14475N, 1.41552E	VII/2012
	9	ES-11	Platja de Llafranc, Callela de Palafrugell	dtto	41.89343N, 3.19391E	VI/2012
	10	ES-10	Platja de Tamariu	dtto	41.91756N, 3.20761E	VI/2012
	11	ES-9	Cala Montgó, L’Escala	dtto	42.10744N, 3.16892E	VI/2012
	12	FR-8	Anse de Paulilles, Paulilles	dtto	42.50236N, 3.12456E	VII/2012
3^rd^	13	FR-20	Les Arnettes	dtto	43.32922N, 5.03849E	VI/2012
	14	FR-7	Baie de Cousse, Sanary-sur-Mer	dtto	43.12054N, 5.77545E	VI/2012
	15	FR-19	Cabasson	dtto	43.09926N, 6.32504E	VI/2012
	16	FR-6	Cap Roux, Saint-Raphaël	dtto	43.45026N, 6.91951E	VI/2012
	17	FR-5	Antibes	dtto	43.55726N, 7.12209E	VI/2012
	18	IT-4	Finale Ligure	dtto	44.17337N, 8.36765E	VI/2012
	19	IT-3	Mulinetto Beach, Cogoleto	dtto	44.38016N, 8.63467E	VI/2012
4^th^	20	HR-37	Neviđane	Adriatic Sea	43.98368N, 15.33831E	IX/2012
	21	HR-38	Dobropoljana	dtto	43.98713N, 15.33295E	IX/2012
	22	HR-39	Žman	dtto	44.00308N, 15.05930E	IX/2012
	23	HR-2	Kukuljar	dtto	43.75960N, 15.63410E	IX/2012
5^th^	24	HR-1	Borak	dtto	42.92236N, 17.34685E	IX/2012 & IX/2016
	25	ME-36	Krimovica	dtto	42.27985N, 18.78738E	IX/2012
	26	ME-35	Sveti Stefan I	dtto	42.25022N, 18.89463E	IX/2012
	27	ME-34	Petrovac	dtto	42.19762N, 18.93726E	IX/2012
	28	ME-33	Crni Rt, Sutomore	dtto	42.13595N, 19.01549E	IX/2012
6^th^	29	AL-31	Orikum I	dtto	40.34226N, 19.40898E	IX/2012
	30	AL-32	Orikum II	dtto	40.35723N, 19.40926E	IX/2012
	31	GR-30	Kalamionas Beach, Kassiopi	Ionian Sea	39.78941N, 19.91542E	IX/2012
	32	GR-29	Kalami	dtto	39.74227N, 19.93443E	IX/2012

^1^ grouping for pyrosequencing, see Materials and Methods

^2^ sequential numbering corresponding to Figure 2 (along the coast from west to east)

^3^ continues from Vohník et al. 2015, 2016 and 2017. AL = Albania, ES = Spain, FR = France, GR = Greece, HR = Croatia, IT = Italy, ME = Montenegro

^4^ according to Notarbartalo di Sciara and Agardy (2010) in Giacoumi et al. (2013)

### Characterisation of *P.oceanica* root mycobionts by 454-pyrosequencing

For 454-pyrosequencing, root samples of the same weight representing individual localities were pooled into six sample sets (Figure 2, Table 1). DNA extraction, PCR amplification and sequencing was conducted as in Vohník et al. (2017). Briefly, after DNA extraction from surface-sterilised *P.oceanica* fine roots conducted using DNeasy Plant Mini Kit (Qiagen), the ITS region of the nrDNA was amplified in a two-step PCR with primers ITS1F/ITS4 (White et al. 1990; Gardes and Bruns 1993) in the first step. One negative control was included in the PCR analyses. From each DNA extract, two independent PCR reactions were run to avoid PCR bias. The obtained PCR products were then pooled, purified and used as a template for the second PCR with tagged ITS1/ITS4 primers. The resulting six samples and one negative control were purified, quantified, equimolarly mixed with other samples from the same 454-pyrosequencing plate and sequenced on the GS Junior platform (Roche).

In total, pyrosequencing yielded 32127 raw sequences which were subsequently processed in the pipeline SEED 2.0.4 (Větrovský and Baldrian 2013). Quality check (min. quality score 25) and denoising yielded 30935 sequences. Sequences shorter than 500 bp were excluded and the data set was trimmed to the 500 bp sequence length. The obtained 15951 sequences were then clustered to molecular OTUs (MOTUs) using UPARSE implementation in USEARCH 8.1.1861 (Edgar 2013) with 97% similarity threshold. Chimeric sequences identified in this step (198) were deleted to prevent diversity overestimation. Also 81 global singletons were removed from the data set. The consensus sequences were constructed for each MOTU using MAFFT v.7.222 alignments (Katoh et al. 2009), based on the most abundant nucleotide at each position. These consensus sequences were then checked for their closest hits by BLAST algorithm using UNITE (Kõljalg et al. 2013) and GenBank (Sayers et al. 2019) as reference databases. Main MOTUs obtained in this study are listed in Table 2.

**Table 2.** List of main MOTUs (with at least 10 sequences) obtained in this study by tag-encoded 454-pyrosequencing.

MOTU #	Number of sequences in each sample set^1^						Total sequences	Closest match in GenBank/UNITE^2^	Identity of the closest match (species hypothesis in UNITE)	Origin/country of the closest match
	1	2	3	4	5	6				
1*	1566	1661	3279	2757	2131	2447	13841	KC412712	Pleosporales sp. MV-2012 (SH215217.07FU)	*Posidoniaoceanica* root/France
2*	59	88	244	0	19	1	411	KC412712	Pleosporales sp. MV-2012 (SH215217.07FU)	*P.oceanica* root/France
5	80	16	0	0	13	12	121	KY859194	* Alternaria alternata *	Black Spot on *Rhodiolarosea*/China
6	0	101	0	0	0	0	101	JX974800	fungal sp. (SH482095.07FU)	polluted estuarine sediment/China
7	17	0	0	30	0	0	47	KY977441	* Pseudopithomyces chartarum *	endophytic in *Sophoramoorcroftiana*/China(?)
10	2	27	0	0	0	0	29	KU869767	*Lobulomyces* sp.	endophytic in *Gracilariopsislemaneiformis*/China(?)
11	0	23	0	0	0	0	23	KX449413	*Lepistanuda* (SH218331.07FU)	fruitbody/France
12	0	0	0	22	0	0	22	GU062266	*Phlebiatremellosa* (SH175372.07FU)	wood of *Alnusincana*/Latvia
13	15	0	0	0	7	0	22	MF435073	* Epicoccum nigrum *	leaves of *Physalisperuviana*/Ecuador
16	7	3	0	9	0	0	19	KF719965	*Lulwoana* sp. (SH174303.07FU)	*P.oceanica* root/Italy
17	5	0	0	14	0	0	19	KY977441	* Pseudopithomyces chartarum *	endophytic in *Sophoramoorcroftiana*/China(?)
18	0	18	0	0	0	0	18	JF449459	Pezizomycotina sp. (SH208929.07FU)	*Fagussylvatica* leaf litter/Austria
19	0	0	0	16	0	0	16	HQ436045	*Malassezia* sp. (SH176394.07FU)	*Axonopuscompressus* soil/Singapore
21	0	0	13	0	0	0	13	KF639790	Pezizomycotina sp. (SH220055.07FU)	photographic material/Slovakia(?)
22	0	0	13	0	0	0	13	KY582119	*Cladosporium* sp.	root of *Nicotianabenthamiana*/Australia
23*	0	0	1	8	1	2	12	KC412712	Pleosporales sp. MV-2012 (SH215217.07FU)	*P.oceanica* root/France
24	0	11	0	0	0	0	11	KC965614	Chytridiomycota sp. (SH486050.07FU)	arctic soil/USA
25	11	0	0	0	0	0	11	UDB019799	*Rhodocollybiabutyracea* (SH209203.07FU)	fruitbody/Estonia

^1^ There were six sample sets representing different parts of the northwest Mediterranean Sea, see Materials and methods, Figure 2 and Table 1

^2^ For details see Suppl. material 1 * MOTUs with closest sequence similarity to the Pleosporales sp. MV-2012 (SH215217.07FU in UNITE) (= *Posidoniomycesatricolor*)

### Isolation and characterisation of *P.oceanica* root mycobionts at the original locality

Root mycobionts were isolated from surface-sterilised terminal fine roots as described in Vohník et al. (2016) except that ten different media, amended with Novobiocin sodium salt (50 mg/L; Sigma-Aldrich, Germany) to prevent growth of bacteria, were used. These included glucose peptone yeast agar (GPYA; glucose 40 g, peptone 5 g, yeast extract 5 g and agar 15 g dissolved in 1 L of deionized water), GPYA + *Posidonia* extract, malt extract (MEA; HiMedia Pvt. Ltd., India), MEA + *Posidonia* extract, MEA with mycological peptone (MEAP; HiMedia), MEAP + *Posidonia* extract, modified Melin-Norkrans medium (MMN; Marx 1969), MMN + *Posidonia* extract, potato dextrose agar (PDA; HiMedia) and PDA + *Posidonia* extract. The *Posidonia* extract was prepared by soaking 200 g of *P.oceanica* leaves, roots, rhizomes and matte at 60 °C in 1 L of seawater for 30 min (Panno et al. 2013), filtrated and 100 mL of the filtrate was mixed with 900 mL of the respective media.

Segments of the surface-sterilised terminal fine roots (ca. 3–5 mm long) were incubated on the surface of the abovementioned solidified media at room temperature in the dark and periodically checked for mycelial growth. There were 50 segments per each medium in two square 25-compartment plastic Petri dishes, i.e. 500 segments in total. The incubation was terminated after ca. 10 months (28^th^ September 2016 – 3^rd^ July 2017) and the obtained isolates were conservatively grouped into several morphotypes using stereomicroscopy and colony characteristics according to Vohník et al. (2016).

### DNA extraction, amplification and Sanger sequencing

DNA was extracted from multiple isolates of each morphotype/medium combination using Extract-N-Amp Plant Kits (Sigma-Aldrich, Germany) following manufacturer’s instructions. Primers used for the amplification of genes and gene regions included: 1) NS7, ITS1F, ITS2 and ITS4 (White et al. 1990; Gardes and Bruns 1993) for the ITS nrDNA, 2) LR0R and LR5 (Vilgalys and Hester 1990; Vilgalys unpublished: www.botany.duke.edu /fungi/mycolab) for the partial nucLSU (D1 and D2 domains), 3) NSSU131 and NS24 (Gargas and Taylor 1992; Kauff and Lutzoni 2002) for the whole nucSSU and 4) fRPB2-5F and fRPB2-7cR (Liu et al. 1999) for the segments 5–7 of the *RPB*2. PCR amplifications were carried out according to the methods described in Vohník et al. (2012). Primers used to sequence the purified PCR products included the amplification primers and nested primers: 1) NSSU897R, NSSU1088 and NS6 (White et al. 1990; Kauff and Lutzoni 2002) for the nucSSU and 2) RPB2-980F and RPB2-1014R (Reeb et al. 2004) for segments 5–7 of the *RPB*2 gene. Automated sequencing was carried out by Macrogen Europe Laboratory (Macrogen Inc., The Netherlands).

The obtained sequences were screened in Finch TV v.1.4.0 (https://digitalworldbiology.com/FinchTV) for possible machine errors, manually edited when needed and subjected to BLAST searches (BLASTn) in GenBank (Altschul et al. 1997). Sequences similar to identical to those previously deposited in GenBank as “Pleosporales sp. MV-2012” (Vohník et al. 2016, 2017) were aligned using ClustalW implemented in BioEdit v.7.1.8 (Hall 1999) to further screen their heterogeneity.

### Sequence alignment and phylogenetic analyses

GenBank accession numbers for ITS, nucLSU, nucSSU and *RPB*2 sequences generated in this study and previously published sequences of the Aigialaceae (Pleosporales, Dothideomycetes) are listed in Suppl. material 2. Homologous nucLSU, nucSSU and *RPB*2 sequences of members of the Aigialaceae were selected from the top-scoring matches using BLASTn and retrieved from GenBank.

The nucLSU, nucSSU and *RPB*2 sequences were manually aligned in BioEdit. The *RPB*2 sequences were transformed into protein sequences maintaining a correct reading frame using the BioEdit programme. This alignment was improved by taking into account the exchangeability of amino acids with similar chemical properties at certain positions. The protein alignment was converted back into a DNA alignment. Single locus data sets for Aigialaceae (nucLSU: 46 sequences/876 characters including gaps; nucSSU: 40/1044; *RPB*2: 24/940) were assessed for conflicts using the 70% reciprocal bootstrap criterion (Mason-Gamer and Kellogg 1996) based on the comparison of the trees obtained with 1000 bootstrap (BS) replicates with RAxML-HPC v.7.0.3 (Stamatakis 2006). Conflict-free datasets were concatenated into a multi-locus alignment (deposited as TreeBASE 24210) that was subjected to a phylogenetic analysis.

Phylogenetic relationships of the Pleosporales sp. MV-2012 were inferred based on the analysis of the combined nucLSU-nucSSU-*RPB*2 sequences of 42 representatives of the Aigialaceae. Four Botryosphaeriales (*Lasiodiplodialignicola*, *Neofusicoccumribis*, *Phyllostictaampelicida* and *Saccharatakirstenboschensis*) were used as an outgroup to root the tree. The first 49, 103 and 123 nt of nucLSU, nucSSU and *RPB*2 at the 5’-end and 480 and 595 nt of nucLSU and nucSSU at the 3’-end, respectively, were excluded from the alignment because of the incompleteness of the majority of sequences. Ambiguous regions were excluded from the alignment. To examine intraspecific variability, a phylogenetic analysis of 17 ITS sequences of the Pleosporales sp. MV-2012 strains and four other members of the Aigialaceae was conducted, with *Astrosphaeriellabambusae* (Pleosporales) selected as an outgroup to root the tree. Due to a long insertion in the ITS1 in all isolates of the Pleosporales sp. MV-2012, a larger part of this sequence was not homologous with the rest of ITS1 sequences of the Aigialaceae. Therefore, the first 334 nt at the 3’-end of ITS1 were excluded and only the remaining 115 nt of ITS1, whole 5.8S and ITS2 were analysed.

The combined dataset was partitioned into three subsets of nucleotide sites (nucLSU, nucSSU, *RPB*2) for which we assumed rate heterogeneity. Maximum Likelihood (ML) and Bayesian Inference (BI) analyses were used to estimate phylogenetic relationships. ML analyses were performed with RAxML-HPC v.7.0.3 with a GTRCAT approximation. Nodal support was determined by non-parametric BS analysis with 1 000 replicates. BI analyses were performed in a likelihood framework as implemented in MrBayes v.3.2.6 (Huelsenbeck and Ronquist 2001) through the CIPRES Science Gateway v.3.3 (http://www.phylo.org) (Miller et al. 2010). For the BI approach, MrModeltest2 v.2.3 (Nylander 2008) was used to infer the appropriate substitution model that best fit the model of DNA evolution. The SYM+G model was selected according to the Akaike information criterion for ITS and all partitions of the Aigialaceae data sets. Two Bayesian searches were performed using default parameters. The B-MCMCMC analyses lasted until the average standard deviation of split frequencies was below 0.01 with trees saved every 1000 generations. The first 25% of saved trees, representing the burn-in phase of the analysis, were discarded. The remaining trees were used for calculating posterior probabilities (PP) of recovered branches. The illustration of phylogenetic relationships is a ML tree.

## Results

### Characterisation of *P.oceanica* root mycobionts by 454-pyrosequencing

The obtained sequences clustered into 61 MOTUs. Read numbers of 13 MOTUs detected in the negative control were subtracted from the read numbers of these particular MOTUs in each of the six samples (if present there), resulting in 14917 sequences in total remaining in the dataset. The most frequent MOTU 1 (13841 sequences in total) was present in all six sample sets (min. 1566, max. 3279 and avg. 2307 sequences per set) and matched with 99.8% similarity and 95.2% coverage with the sequence KC412712 (see Table 2) derived from the Pleosporales sp. MV-2012 (UNITE species hypothesis SH215217.07FU) isolate P15 previously obtained from *P.oceanica* surface-sterilized root segment collected at one of the localities also investigated in this study (France, Baie de Cousse, Sanary-sur-Mer; Table 1) (Vohník et al. 2016). Twenty one other MOTUs, including the second most frequent MOTU 2 (411 sequences in total) present in five sample sets (min. 1, max. 244 and avg. 69 sequences per set) matched with sequences representing the same species hypothesis SH215217.07FU (Suppl. material 1). When counting all these 22 MOTUs together, they comprised 14334 sequences, i.e. 96% of all sequences. In contrast, the two MOTUs (MOTU 16 and MOTU 48) with a close match to mycobionts from the family Lulworthiaceae were represented only by 19 and four sequences and found only in three and two sample sets, respectively (Table 2, Suppl. material 1). The third most frequent MOTU 5 represented the ubiquitous ascomycete *Alternariaalternata* and the sequences of the fourth most frequent MOTU 6 matched with an undescribed fungus (UNITE species hypothesis SH482095.07FU) from Chinese polluted estuarine sediment. All other MOTUs had each less than 100 sequences in total and represented various Ascomycota, Basidiomycota and Chytridiomycota from mostly terrestrial habitats (many as plant endophytes) of worldwide distribution (see Table 2, Suppl. material 1).

### Isolation and characterisation of *P.oceanica* root mycobionts at the original locality

In total, we obtained 130 fungal mycelial isolates, i.e. 26% of the original 500 surface-sterilised root segments yielded mycelial isolates. There were no obvious effects of the isolation media on the mycelial isolate recovery except that the most isolates (i.e. 26) were obtained on PDA + *Posidonia* extract followed by PDA (23 isolates). With respect to recovery of the Pleosporales sp. MV-2012, the most efficient media were MMN and PDA + *Posidonia* extract with 55.6% and 53.8%, respectively. MMN and PDA were the two isolation media used in the first study to report the Pleosporales sp. MV-2012 from *P.oceanica* roots (Vohník et al. 2016).

Most of the obtained isolates were conservatively grouped into two dominant morphotypes, i.e. “Black” (62 isolates) and “Yellow” (38), where the former was morphologically identical to the Pleosporales sp. MV-2012 and the latter roughly corresponded to the Lulworthiales sp. MV-2012 described in Vohník et al. (2016, 2017). Approximately one third of the Black isolates, all the Yellow isolates and all remaining isolates were subjected to DNA extraction, amplification and sequencing which led to identification of 91 isolates. Since all sequenced Black isolates yielded high-quality sequences matching the Pleosporales sp. MV-2012, it was likely that also the rest of the Black isolates (i.e. those that were not selected for sequencing) belonged to this species, i.e. in total 112 isolates (ca. 86 %) were identified. Out of the identified isolates, the Pleosporales sp. MV-2012 represented 54.5%. All the Yellow isolates belonged to the Lulworthiales and matched the Lulworthiales sp. MV-2012, the Lulworthiales sp. MV-2012B (see Vohník et al. 2017) and *Lulwoana* sp. The remaining unidentified isolates either failed to amplify or produced mixed sequences suggesting their non-axenic status (data not shown).

After prolonged cultivation, several Pleosporales sp. MV-2012 isolates started to produce submerged mycelium and two of them were successfully transferred and maintained on potato carrot agar (PCA). These isolates were used for the phylogenetic analysis and the formal description of the dominant *P.oceanica* root mycobiont (see below).

### Phylogenetic analysis

**Figure 3.**
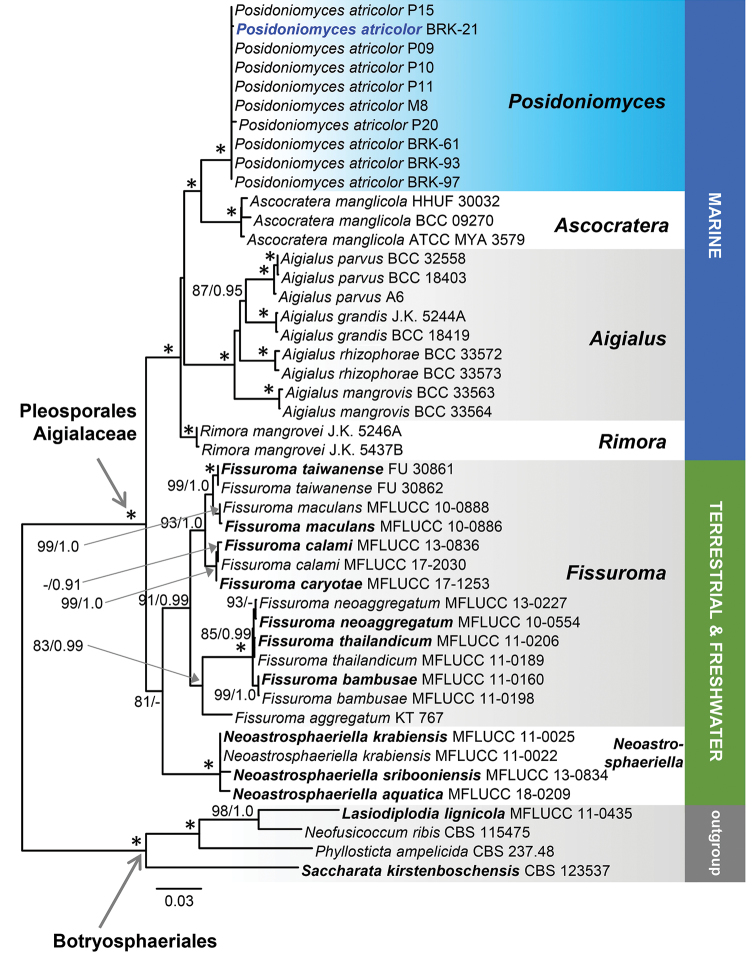
Phylogram generated from maximum likelihood analysis based on combined nucLSU, nucSSU and *RPB2* sequence data for *Posidoniomycesatricolor* and the Aigialaceae. Species names given in bold are type species. The ex-type of the taxonomic novelty is in bold and blue. An asterisk (*) indicates branches with ML BS = 100% and PP values = 1.0. Branch support of nodes ≥ 70 % ML BS and ≥ 0.90 PP is indicated above or below branches.

A previous phylogenetic analysis of nucLSU sequences of members of nine families of the Pleosporales (Vohník et al. 2016) positioned the Pleosporales sp. MV-2012 in the Aigialaceae. In line with these results, we performed a subsequent analysis and phylogenetic relationships were inferred based on the combined nucLSU-nucSSU-*RPB*2 sequences of 10 isolates of the Pleosporales sp. MV-2012 and 32 additional isolates representing 17 species of five genera (*Ascocratera*, *Aigialus*, *Rimora*, *Fissuroma* and *Neoastrosphaeriella*) of the Aigialaceae. The full data set consisted of 2860 characters and 936 unique character sites. There were no differences in the topologies of trees generated from BI and ML analyses. In the ML tree (Figure 3), members of the Aigialaceae (100% ML BS/1.0 PP) formed two subclades defined by ecology. One subclade (81/0.84) contained taxa known only from terrestrial and freshwater habitats, i.e. *Fissuroma* and *Neoastrosphaeriella*. The other subclade (100/1.0) contained marine saprobic species of *Ascocratera*, *Aigialus* and *Rimora* occurring on mangroves growing in estuarine environments and also a new marine lineage represented by the Pleosporales sp. MV-2012 associated with the roots of the seagrass *P.oceanica* and described as a new genus *Posidoniomyces* below.

**Figure 4.**
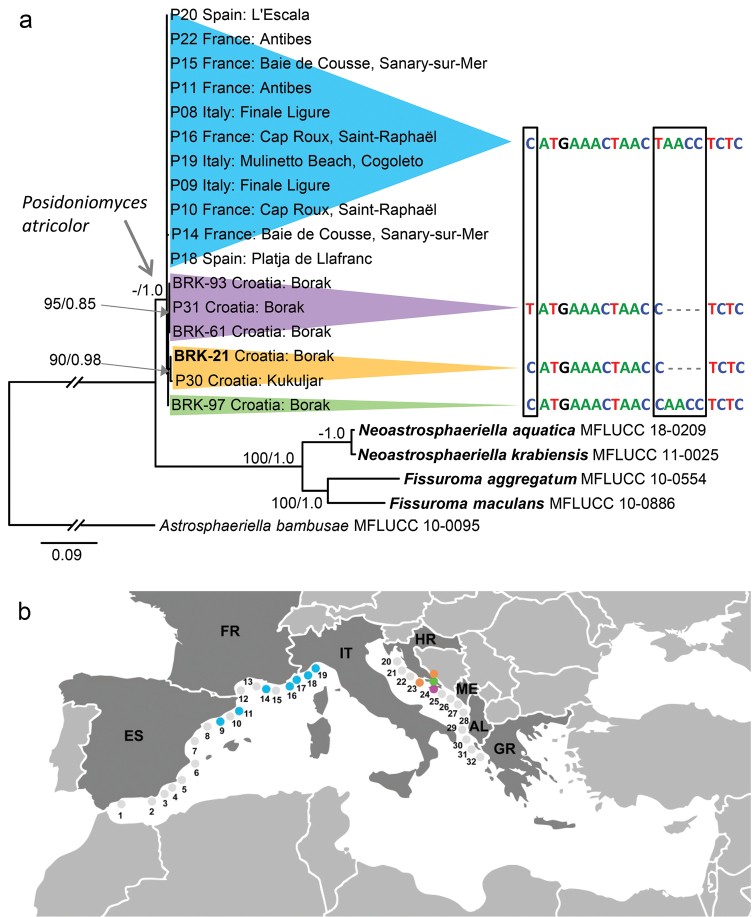
Phylogram and map showing a distribution pattern of *Posidoniomycesatricolor*. **a** Phylogram generated from maximum likelihood analysis based on ITS sequence data for *Posidoniomycesatricolor* and representatives of the Aigialaceae**b** map of the Mediterranean Sea with our 32 sampling sites. Sites in blue, orange, violet and green colour indicate locations of *P.atricolor* strains with corresponding mutations in ITS2 sequences.

The second analysis was based on ITS (partial ITS1, 5.8S and ITS2) sequences of 17 isolates of *P.atricolor* from nine localities in Croatia, France, Italy and Spain and additional four and only available ITS sequences of representatives of the Aigialaceae, *Fissuroma* and *Neoastrosphaeriella*. The data set consisted of 494 characters and 194 unique character sites. The topologies of trees from BI and ML analyses were identical. The ML tree is shown in Figure 4. *Posidoniomyces* forms monophyletic clade (49/1.0) with four subclades, which correspond to several indels in the ITS2. These changes in the primary sequence characterise populations of *P.atricolor* and their distribution pattern on the north-west coast of France and Spain and north-central part of the Adriatic coast of Croatia.

### Taxonomy

#### Posidoniomyces

Taxon classificationFungiPleosporalesAigialaceae

Vohník & Réblová gen. nov.

MB830266

##### Diagnosis.

In vivo, colonisation pattern of host roots resembles colonisation by the so-called dark septate endophytes (DSE) ubiquitous in the roots of most terrestrial plants. However, the dark septate hyphae and microsclerotia of *Posidoniomyces* never colonise vascular tissues of the host roots and are mostly confined to the hypodermis.

##### Type species.

*Posidoniomycesatricolor* Vohník & Réblová

##### Etymology.

Named after the host seagrass *Posidoniaoceanica* and *myces* (Greek), meaning fungus.

##### Description.

Root mycobiont of the dominant and endemic Mediterranean seagrass *Posidoniaoceanica*. In vivo, hyphae brown, septate, forming intracellular microsclerotia in the hypodermis of the terminal fine roots and finger-like pseudoparenchymatous net on the surface of these roots, i.e. a colonisation pattern resembling the DSE association ubiquitous in the roots of terrestrial plants. In vitro, two distinct colonial morphotypes named compact and mycelial (often with aerial hyphae) are consistently formed. Colonies brown, mycelium composed of septate, hyaline, subhyaline to pigmented hyphae with intercalary, terminal, rarely lateral, one-celled globose, subglobose to ellipsoidal swellings that are prominent especially on the surface of the compact colonies. Sexual state unknown.

#### Posidoniomyces atricolor

Taxon classificationFungiPleosporalesAigialaceae

Vohník & Réblová sp. nov.

MB830267

Figs 5 , 6

##### Typification.

CROATIA. Dubrovnik-Neretva County: Potomje, Borak (42.92236N, 17.34685E), isolated from a surface-sterilised healthy-looking terminal root of *Posidoniaoceanica*, 28 Sep 2016, M.Vohník & O.Borovec BRK-21 (holotype: PRA-15294!, dried culture – compact morphotype from a surface-sterilised root segment; isotype: PRA-15295!, dried culture – mycelial morphotype derived from the original compact colony).

##### Etymology.

*Atricolor* (L), meaning black, dark coloured, referring to the dark pigmented hyphae.

##### Description in culture.

*Mycelial colonial morphotype*: Colonies on PCA 6–8 mm in diameter in 3 mo, circular, convex, appearing woolly, margin entire, aerial mycelium abundant, densest at the centre, cobwebby towards the margin, white to grey with a pale brown zone at the margin, colony surface with a dark brown hue formed by substrate mycelium and released pigment; reverse brown. *Compact colonial morphotype*: Colonies on PCA 5–6 mm in diameter in 8 mo, irregular, pulvinate, deeply furrowed, appearing mucoid-waxy to faintly floccose, of a “cartilage” consistency, become hollow upon aging, margin lobate, aerial mycelium scant, hyaline to pale brown, colony surface dark brown; reverse dark brown. Compact colonies, which are formed in vitro on sterilised roots of *P.oceanica*, become irregular in shape, folded and furrowed in an almost cerebriform pattern, cacao brown, ca. 5–6 mm long on the longest side after several months of cultivation. *Hyphae* hyaline to pale brown, septate, smooth-walled and 2–3(–3.5) µm wide, often with terminal, intercalary, rarely with lateral, one-celled, thick-walled globose, subglobose to ellipsoidal swellings 10–14 µm wide; hyphae frequently protrude from these swellings and continue growing. Surface of the compact colonies covered by hyaline to subhyaline, smooth-walled hyphae with terminal, capitate swellings. Chlamydospores, conidiogenous cells or conidia, ascomatal initials and ascomata not observed.

##### Description in vivo.

In vivo *hyphae* pigmented, septate, smooth-walled and (2–)3–4(–5) µm wide, colonising root cells of the host and/or forming an extraradical hyphal sheath, i.e. a finger-like pseudoparenchymatous net on the root surface. *Microsclerotia* intracellular, melanised, round or elongated and 8–10(–17) µm wide, present in the *P.oceanica* root hypodermis. Intracellular hyphae also infrequently occur in the rhizodermis.

##### Specimens examined.

Croatia. Dubrovnik-Neretva County: Potomje, Borak (42.92236N, 17.34685E), isolated from surface-sterilised healthy-looking terminal roots of *P.oceanica*, 28 Sep 2016, M.Vohník & O.Borovec BRK-11 (PRA-15296); ibid., BRK-25 (PRA-15298); BRK-34 (PRA-15297); BRK-60 (PRA-15300); BRK-61 (PRA-15293); BRK-76 (PRA-15302); BRK-87 (PRA-15299); BRK-93 (PRA-15301), BRK-97 (PRA-15303). Croatia. Split-Dalmatia County: Palagruža archipelago, Gangaro Island I (43.8639N, 15.4341E), isolated from a surface-sterilised healthy-looking terminal root of *P.oceanica*, 3 September 2012, M.Vohník & O.Borovec M8. France. Provence-Alpes-Côte d’Azur Region: Var Department, Saint-Raphaël, Cap Roux (43.45026N, 6.91951E), isolated from a surface-sterilised healthy-looking terminal root of *P.oceanica*, 17 June 2012, M.Vohník P10. France. Provence-Alpes-Côte d’Azur Region: Alpes-Maritimes Department, Antibes (43.55726N, 7.12209E), isolated from a surface-sterilised healthy-looking terminal root of *P.oceanica*, 18 June 2012, M.Vohník P11. France. Provence-Alpes-Côte d’Azur Region: Var Department, Sanary-sur-Mer (43.12054N, 5.77545E), isolated from a surface-sterilised healthy-looking terminal root of *P.oceanica*, 19 June 2012, M.Vohník P15. Italy. Liguria Region: Savona Province, Gulf of Genoa, Finale Ligure (44.17337N, 8.36765E), isolated from a surface-sterilised healthy-looking terminal root of *P.oceanica*, 17 June 2012, M.Vohník P09. Spain. Girona Province: L’Escala (42.10744N, 3.16892E), isolated from a surface-sterilised healthy-looking terminal root of *P.oceanica*, 18 June 2012, M.Vohník P20.

##### Habitat and distribution.

Root mycobiont of the dominant and endemic Mediterranean seagrass *Posidoniaoceanica*. So far known only from the NW Mediterranean Sea.

##### Notes.

**Figure 5.**
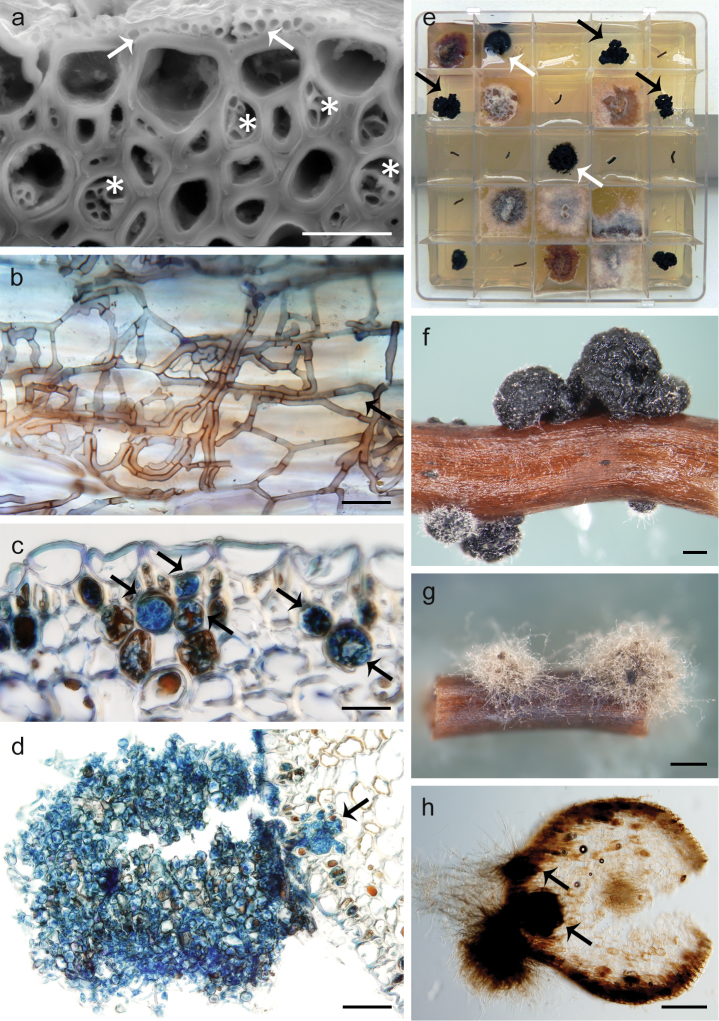
In vivo root colonisation pattern and in vitro cultural aspects of *Posidoniomycesatricolor*. **a** In vivo colonisation on the root surface (arrows) and in the hypodermis (asterisks) of *P.oceanica***b**DSE colonisation on the root surface **c** germinating microsclerotia stained with trypan blue (arrows) **d** compact colony developed from microsclerotia (arrow) **e** surface-sterilised root segments yielding *P.atricolor* compact colonies (black arrows), sometimes with substrate mycelium (white arrows) **f** compact colonial morphotype **g** mycelial colonial morphotype **h** mycelial morphotype developing from microsclerotia (arrows) in transversal section. Scale bars: 20 μm (**a, b**), 50 μm (**c**), 100 μm (**d**), 200 μm (**f, h**), 500 μm (**g**).

**Figure 6.**
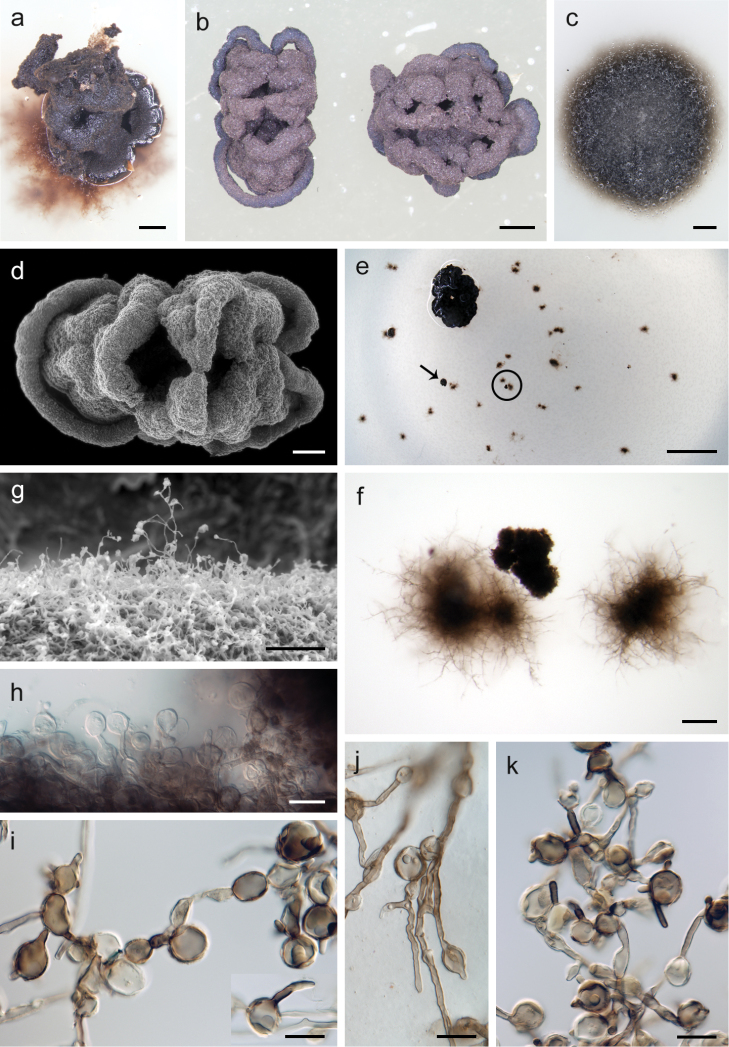
Colonial morphotypes of *Posidoniomycesatricolor* in vitro (type isolate BRK-21). **a** Compact morphotype with substrate mycelium **b, d** compact colonies with a cerebriform pattern **c** colony of *P.atricolor* on PCA **e** rhizoidal and compact (arrow) daughter colonies on PCA washed with sterile tap water **f** detail of the colonies encircled in **e**; **g, h** terminal capitate swellings on the surface of compact colonies **i–k** conspicuous swellings on aerial mycelium. Scale bars: 500 μm (**a, d**), 1000 μm (**b, c**), 5 mm (**e**), 200 μm (**f**), 100 μm (**g**), 20 μm (**h**).

Both colonial morphotypes, named compact and mycelial, appeared on surface-sterilised root segments of *P.oceanica* and after inoculation also on solid agar media but the compact colonies with the cerebriform pattern formed only on the original root segments. All examined colonies of *P.atricolor* emerging from the original root segments developed from melanised microsclerotia formed exclusively intracellularly in the *P.oceanica* hypodermis (Figure 5d, h). The mycelial morphotype was observed on MMN and PCA, while compact colonies were formed on PDA and PCA (Vohník et al. 2016; this study). When the surface of a colony exhibiting the compact colonial morphotype was washed regularly with sterile tap water, fragments of hyphae were released to form minute daughter colonies (Figure 6e). These daughter colonies were either of a rhizoidal form composed of substrate mycelium and continued to develop the mycelial morphotype or they assumed the compact colony character from the beginning (Figure 6f). A new hypha was often formed through the globose swelling, regardless of its position on the hypha (Figures 6i–k).

## Discussion

The microscopic screening of *Posidoniaoceanica* root fungal colonisation confirms that the recently described DSE association (Vohník et al. 2015) formed by the Pleosporales sp. MV-2012 (Vohník et al. 2016) and introduced as *Posidoniomycesatricolor* in this study, is present at all investigated localities. The tag-encoded 454-pyrosequencing of fungal DNA extracted from surface-sterilised *P.oceanica* root segments confirms the dominance of this fungus in the root mycobiota of the dominant seagrass in the NW Mediterranean Sea. Our analysis of combined DNA sequences of nuclear ribosomal and protein-coding loci confirms the placement of *P.atricolor* in the Aigialaceae (Pleosporales, Dothideomycetes) and suggests an independent marine biotrophic lineage.

The root-symbiotic *Posidoniomyces* is related to mostly saprobic lignicolous marine fungi from estuarine environments colonising wood and roots of mangroves growing in tropical regions of both Eastern and Western Hemispheres, a situation resembling, at least to some extent, the relationship of the ubiquitous terrestrial root-symbiotic *Rhizoscyphusericae* aggregate to saprobic fungi from the genus *Hyaloscypha* (Fehrer et al. 2019). Because mycorrhizal fungi from the *R.ericae* aggregate have significant saprobic abilities (Martino et al. 2018), they can decompose recalcitrant peat and exchange mineral nutrients (especially nitrogen) for the host photosynthetically bound carbon. Since *P.oceanica* often grows on thick layers of recalcitrant peat-like matte (Figure 1b, c), which typically stores large amounts of organically-bound nutrients (Fourqurean et al. 2012) directly unavailable to plants (Read 1991), it is tempting to speculate about the possible role of *P.atricolor* in mineral nutrition of the dominant Mediterranean seagrass (also see Borovec and Vohník 2018; Kolátková and Vohník 2019). On the other hand, genomes of DSE fungi typically combine saprobic and pathogenic traits (Schlegel et al. 2016; Knapp et al. 2018) and effects of root endophytes on host plant fitness vary along the parasitism-mutualism continuum (Newsham 2011; Mayerhofer et al. 2013). Thus, although the specific association with *P.atricolor* is omnipresent in *P.oceanica* at all so far investigated localities, to date there is no solid proof that it is of any benefit to the seagrass.

The Aigialaceae (Suetrong et al. 2009) was erected for marine ascomycetes characterised by fisstunicate asci with a non-amyloid apex and a ring-like apical apparatus containing septate or muriform ascospores with a gelatinous sheath or cap, trabeculate hamathecium and non-stromatic, carbonatious to coriaceous, non-papillate ascomata. Additionally, *Fissuroma* and *Neoastrosphaeriella* occurring on bamboo, palms and flowering plants in terrestrial and freshwater environments were added to the family by Liu et al. (2011). The asexual morphs of marine species are generally unknown. The asexual morph of *Fissuroma* was reported as coelomycetous, pleurophomopsis-like (Tanaka and Harada 2005; Liu et al. 2011). Axenic cultures of *P.atricolor* remained sterile and two colonial morphotypes, named compact and mycelial, were consistently formed originating from the primary source. Although the presence of both morphotypes on PCA, a low sugar content medium, would suggest that the mode of nutrition does not influence the colony appearance, the absence of one or the other morphotype on MMN and PDA may indicate that the nutrition mode could play a role to some extent. When a compact colony was regularly washed with sterile tap water, a number of daughter colonies were formed all over the agar plate (Figure 6e), suggesting that liquid culture might be an efficient way for producing larger quantities of *P.atricolor* mycelium. These colonies usually assumed the form of a miniature rhizoidal-like colony (Figure 6f) formed mainly by submerged mycelium or they formed the well-distinguishable compact colonial morphotype directly. It is probable that the capitate swellings protruding above the surface of the compact colonies (Figure 6g, h) together with hyphal fragments act as propagules in the absence of conidia and ensure the dispersal of the fungus in a simulated environment. These terminal, intercalary and sometimes lateral mostly globose swellings resemble intercalary conidia of species of *Knufia*, e.g. *K.perforans*, formed on elongated and monilioid hyphae (Tsuneda et al. 2011). However, *P.atricolor* compact colonies have never been observed in vivo directly on *P.oceanica* roots and it is thus unknown whether the capitate swellings form and act as propagules also under natural conditions.

The Dothideomycetes include several marine genera that usually do not form an asexual state and are distributed in several orders, i.e. Capnodiales, Dothideales, Hysteriales, Jahnulales, Patellariales and Pleosporales, or *incertae sedis* lineages (Suetrong et al. 2009). They include mainly taxa thriving in intertidal zone on a variety of substrates of mangroves in tropics or less frequently on salt marsh plants in temperate regions. Other marine Dothideomycetes can occur as parasites or possible endophytes of seagrasses or marine macroalgae and are completely submerged. The omnipresence and dominance of *P.atricolor* in the roots of *P.oceanica* suggests a close symbiotic relationship with the dominant Mediterranean seagrass, a trait so far unparalleled in other Dothideomycetes. At the same time, to our knowledge, the characteristic DSE colonisation pattern of *P.atricolor* has never been observed in any other seagrass species, suggesting its specificity for *P.oceanica* (also see Discussion in Vohník et al. 2015).

The analysis of all available *P.atricolor* ITS sequences (Vohník et al. 2016, 2017; this study) revealed several aspects that may connect with their geographic distribution and possibly also the symbiotic lifestyle. The ITS1 region of *P.atricolor* contains ca. 294 nt long insertion near the 5’-end when compared to ITS1 of other members of the Aigialaceae. Only four species of the whole family have their ITS sequences available; the ITS1 varies between 151–168 nt in *Fissuroma* (*F.maculans*, *F.neoaggregatum*) and between 186–201 nt in *Neoastrosphaeriella* (*N.aquatica*, *N.krabiensis*), compared to 445 nt in *P.atricolor*. When the ITS1, 5.8S and ITS2 of *P.atricolor* were checked for closest hits by the BLAST search in GenBank, the closest relatives for the 5.8S region were members of the Aigialaceae and other taxa of the Pleosporales; however, no close hits were revealed for ITS1 and ITS2. Since the ITS region was amplified and sequenced as a part of the whole nuc18S region with several forward and reverse primers, it is unlikely that this divergence was caused by PCR or sequencing errors. ITS is a rapidly evolving region where numerous insertions and deletions occur. Considering the probably obligate symbiotic lifestyle of *P.atricolor* in the host roots, the long insertion in ITS1 and high divergence in ITS2 sequences may be a result of co-evolution of both partners, higher gene flow rate and possibly horizontal gene transfer resulting in genetic mismatches in the fungal partner (Saikkonen et al. 2004, 2010; also see Kolařík and Vohník 2018). However, outside the Aigialaceae, the ITS1 region can be much longer, for example in *Astrosphaeriellabambusae*, the outgroup, it is 445 nt long. On the other hand, the length of the ITS2 region is comparable between *P.atricolor* (188–192 nt) and other members of the Aigialaceae (156–163 nt).

Although the ITS sequences of all *P.atricolor* isolates are nearly identical (99.87–98.99% identity between the type strain BRK-21 and other isolates), they differ in up to six indels near the 5’-end of the ITS2. These site mutations can be used to some extent to characterise different populations of *P.atricolor* (Figure 4). Only strains which could be compared morphologically, i.e. those successfully derived from *P.oceanica* surface-sterilised root segments into axenic culture (Vohník et al. 2016; this study), were analysed. Their colony characters and colonisation pattern in the roots of the host were identical. At the ITS2 sequence level, we could distinguish populations from the north-west regions of the Mediterranean (France, Spain) and those from the north-central part of the Adriatic coast (Croatia). Moreover, the Croatian population from Borak (locality HR-1; Table 1) seems to be a source of several mutations. Further screening of *P.oceanica* root mycobiota outside the NW Mediterranean is apparently needed to fully elucidate the usefulness of ITS sequences for distinguishing geographically different populations of *P.atricolor*.

Although it is a significant producer of biomass and an important source of decomposing organic matter in the sea and adjacent habitats, the mycobiota of *P.oceanica* has been studied only by a few authors (e.g. Kohlmeyer 1963; Cuomo et al. 1985; Panno et al. 2013; Gnavi et al. 2014; Vohník et al. 2016, 2017; this study), with differing results. Most significantly, no study prior to Vohník et al. (2016) reported *P.atricolor* in the mycobiota of the dominant Mediterranean seagrass. This is probably due to the manner of material sampling and isolation procedure, i.e. direct isolation from decaying plant matter vs. serially washed or surface-sterilised parts of living plants, the former often leading to detection of fast-growing surface-dwelling saprobes in contrast to isolation of true endophytes (Sieber 2002; also see Discussion in Vohník et al. 2016, 2017). Indeed, apart from *P.atricolor* and the obligate marine Sordariomycetes (*Corollosporamarina* and *C.intermedia* in Microascales, *Lulwoana* sp. and *Lulworthia* sp. in Lulworthiales and *Papulasporahalima* incertae sedis) and Dothideomycetes (*Halotthiaposidoniae*, *Pontoporeiabiturbinata* and several other genera in Pleosporales), majority of the fungi reported from *P.oceanica* are asexually reproducing ubiquitous fungi (Panno et al. 2013).

The distribution pattern of *P.oceanica* mycobiota in leaves, rhizomes, roots and matte is affected by various environmental parameters, presence of growth-inhibiting substances in leaves or antagonistic organisms and may be also influenced by the season (Cuomo et al. 1985; Panno et al. 2013; Gnavi et al. 2014). However, no detailed data are yet available for the dominant root mycobiont *P.atricolor*, except that it seems to be restricted to *P.oceanica* roots. Mycorrhizal fungi form often vigorous extraradical mycelium penetrating the substrate far beyond the rhizosphere, thus forming the mycorrhizosphere (i.e. a volume of soil under a combined influence of the root and the emerging fungal hyphae) (Linderman 1988). The mycorrhizosphere significantly enlarges the volume of the substrate available for mycorrhizal nutrient uptake and in a way defines individual mycorrhizal types. It would therefore be interesting to screen the volume and enzymatic activity of the *P.atricolor* extraradical mycelium (if existing) to decide more precisely about the mode of the interaction between the dominant Mediterranean seagrass and its dominant root mycobiont.

## Conclusions

This study confirms at an unprecedented scale that the diversity of the root mycobiota of the dominant Mediterranean seagrass is relatively narrow and dominated by a single pleosporalean fungus so far not known from any other hosts or environments. This fungus is introduced here as a new genus and species *Posidoniomycesatricolor* and resides as an independent marine biotrophic lineage in the Aigialaceae. The characteristic colonisation pattern of *P.atricolor* in *P.oceanica* roots has not been reported in any other seagrass and resembles colonisation by DSE fungi which are ubiquitous in terrestrial roots. Further research is needed on the distribution and genetic variability (especially ITS sequences) of *P.atricolor* in the rest of the Mediterranean Sea (i.e. Eastern Mediterranean Basin, the coast of North Africa). Additionally, given the uniquely discontinuous distribution area of the genus *Posidonia* (Green and Short 2003), targeted research on the root mycobiota of its Australian species would be of a special evolutionary significance.

## Supplementary Material

XML Treatment for Posidoniomyces

XML Treatment for Posidoniomyces atricolor

## Supplementary materials

Supplementary material 1 Characteristics of fungal MOTUs obtained from surface sterilized *Posidoniaoceanica* roots in this study Data type: species data This dataset is made available under the Open Database License (http://opendatacommons.org/licenses/odbl/1.0/). The Open Database License (ODbL) is a license agreement intended to allow users to freely share, modify, and use this Dataset while maintaining this same freedom for others, provided that the original source and author(s) are credited.Martin Vohník, Ondřej Borovec, Zuzana Kolaříková, Radka Sudová, Martina Réblová

Supplementary material 2 A list of fungi, isolate information and new sequences determined for this study (in bold) and additional sequences retrieved from GenBank Data type: species data This dataset is made available under the Open Database License (http://opendatacommons.org/licenses/odbl/1.0/). The Open Database License (ODbL) is a license agreement intended to allow users to freely share, modify, and use this Dataset while maintaining this same freedom for others, provided that the original source and author(s) are credited.Martin Vohník, Ondřej Borovec, Zuzana Kolaříková, Radka Sudová, Martina Réblová
